# Improving the Acceptability of Human Papillomavirus Vaccines Among Men Who Have Sex With Men According to the Associated Factors: A Systematic Review and Meta-analysis

**DOI:** 10.3389/fphar.2021.600273

**Published:** 2021-03-24

**Authors:** Yang Zhao, Xiaoli Xin, Huiwen Deng, Junjie Xu, Wenjia Weng, Ming Zhang, Juan Li, Yanqing Gao, Xiaojie Huang, Cuie Liu

**Affiliations:** ^1^ Department of Dermatology, Beijing Youan Hospital, Capital Medical University, Beijing, China; ^2^ Shenyang Sixth People’s Hospital of Shenyang, Shenyang, China; ^3^ The First Hospital of China Medical University, Shenyang, China; ^4^ Key Laboratory of AIDS Immunology of Liaoning Province, the First Affiliated Hospital of China Medical University, Shenyang, China; ^5^ Center for Infectious Diseases,Beijing Youan Hospital, Capital Medical University, Beijing, China; ^6^ Candidate Branch of National Clinical Research Center for Skin Diseases, Beijing, China

**Keywords:** human papilloma virus, HPV vaccine, men who have sex with men, acceptability, systematic review

## Abstract

**Objectives**: To investigate the acceptability of human papillomavirus (HPV) vaccination among men who have sex with men (MSM) and its associated factors.

**Methods**: We searched studies written in English in PubMed, EMBASE, and Web of Science with no geographical or time restrictions. We evaluated the quality of the included literature. We calculated the pooled acceptability and performed meta-analysis of selected studies, including factors associated with the acceptability among MSM, using Review Manager (v5.3).

**Results**: The acceptability among the 15 studies (*n* = 8,658) was 50% (95% CI: 0.27–0.72). The meta-analysis of seven articles (*n* = 4,200) indicated that having a college or higher degree (OR = 1.62, 95% CI: 1.35–1.95), disclosure of sexual orientation to healthcare professionals (HCPs; OR = 2.38, 95% CI: 1.47–3.86), vaccination with at least one dose for hepatitis A or B (OR = 2.10, 95% CI: 1.42–3.10), awareness of HPV (OR = 1.85, 95% CI: 1.21–2.83), knowledge of HPV (SMD = 0.28, 95% CI: 0.16–0.39), perceived susceptibility to HPV infection (SMD = 0.31, 95% CI: 0.11–0.50), and perceived severity of HPV-related disease (SMD = 0.40, 95% CI: 0.28–0.51) can promote acceptance of HPV vaccines. Meanwhile, people who have had unprotected anal sex or have more sex partners tend to have low acceptance of HPV vaccines.

**Conclusions**: HPV education should be actively promoted according to the factors that influence the acceptability of HPV vaccines among the MSM population. HPV education should be especially aimed at people with low academic qualifications and people with risky sexual behaviors, and should emphasize the aspects of susceptibility to and severity of HPV-related disease. More intervention trials should be conducted to increase the credibility of the results.

## Introduction

Human papillomavirus infection, a common sexually transmitted infection, is one of the main causes of anal, cervical, vulvar, vaginal, and penile cancer (Centers for Disease Prevention and Control Human Papillomavirus (HPV), 2019). In addition to women, with whom HPV-related disease are widely associated, men who have sex with men bear a much heavier disease burden compared with the heterosexual population (England PH, 2018). The prevalence of HPV-16, which is strongly associated with anal and penile cancer, is much higher among MSM than among men who have sex with women (MSW). The annual incidence of anal cancer among MSM is nearly 20 times greater compared with MSW (Nyitray et al., 2015; Park et al., 2015; Gerend et al., 2016).

The United States Food and Drug Administration officially approved the first HPV vaccine in 2006. At present, 115 countries have introduced HPV vaccination programs, most of which are female oriented, with the propose of protecting women from cervical cancer (Arie, 2019). Studies have confirmed the role of HPV vaccines in preventing HPV infection, anogenital warts, and cervical intraepithelial neoplasia grade 2+, and HPV vaccination programs for women have shown herd effects (Drolet et al., 2019). However, MSM are unlikely to benefit from herd effects because their sex partners are not vaccinated (Zhang et al., 2017; Nadarzynski et al., 2018). For example, Australian that implemented a women-oriented HPV vaccination program have reported a 93% and an 82% decline in anogenital warts in women (<21 years old) and in unvaccinated heterosexual men, respectively, but no significant decline among MSM (Read et al., 2011; Ali et al., 2013; Zhang et al., 2017).

National HPV vaccination programs for MSM have been proven as feasible and cost effective (England PH, 2018). In 2011, the Advisory Committee on Immunization Practices recommended HPV vaccination through age 26 years for MSM and for immunocompromized persons (including those with HIV infection) if not vaccinated previously (Petrosky et al., 2015). The United Kingdom. began its national HPV vaccination program for MSM in April 2018.

However, the coverage of targeted MSM HPV vaccination has raised concerns. It is hypothesized that only with at least 70% uptake rate of HPV vaccine can prevention effects similar to that in women be observed in MSM (Zhang et al., 2017). In 2014, the uptake rate was 17.2% among MSM aged 18–26 years in the United States (Chow et al., 2015; Oliver et al., 2017; McGrath et al., 2019). Although the rate has increased, the coverage in 2017 (37.6%) remains far from the 2020 target of Healthy People (80%) (Loretan et al., 2019).

In improving the coverage of HPV vaccination, it is necessary to determine the acceptability of vaccination and to investigate the factors that affect such acceptance. We conducted a meta-analysis on these points, aiming to provide more evidence that can serve as reference on this issue.

## Materials and Methods

### Search Strategy

We registered the protocol for the article on INPLASY in advance (INPLASY202070129). We report our methods in accordance with the Meta-analyses Of Observational Studies in Epidemiology Checklist. The credentials of the two investigators are indicated in the author list. We searched EMBASE, Web of Science and PubMed for titles or abstracts that use combinations of the following key words (“gay,” “MSM,” “bisexual men,” or “men who have sex with men”) and (“human papillomavirus vaccine,” “human papillomavirus vaccination,” “HPV vaccine,” or “HPV vaccination”) and (“acceptance,” “acceptability,” “attitudes,” or “perceptions”). The last search date was July 31, 2020. We used EndNote to search for duplicate documents and insert references. We also searched the reference lists of the selected studies. We attempted to contact the authors of the articles of which the full text could not be obtained but failed.

We searched studies written in English in the three databases with no geographical or time restrictions. Studies that met the following criteria were included (Centers for Disease Prevention and Control Human Papillomavirus (HPV), 2019): quantitative original studies (England PH, 2018); investigation on the MSM population (Gerend et al., 2016); results include acceptability (mean scores of scales or percentages) or factors influencing acceptability, such as sociodemographic characteristics and behavioral risk indicators. Studies that investigated people who have been vaccinated were excluded. We also excluded studies that only reported the odds ratios (ORs) or standard mean difference (SMD) but not the number of people who accepted vaccination and those who did not in the two groups with or without influencing factors.

**FIGURE 1:**
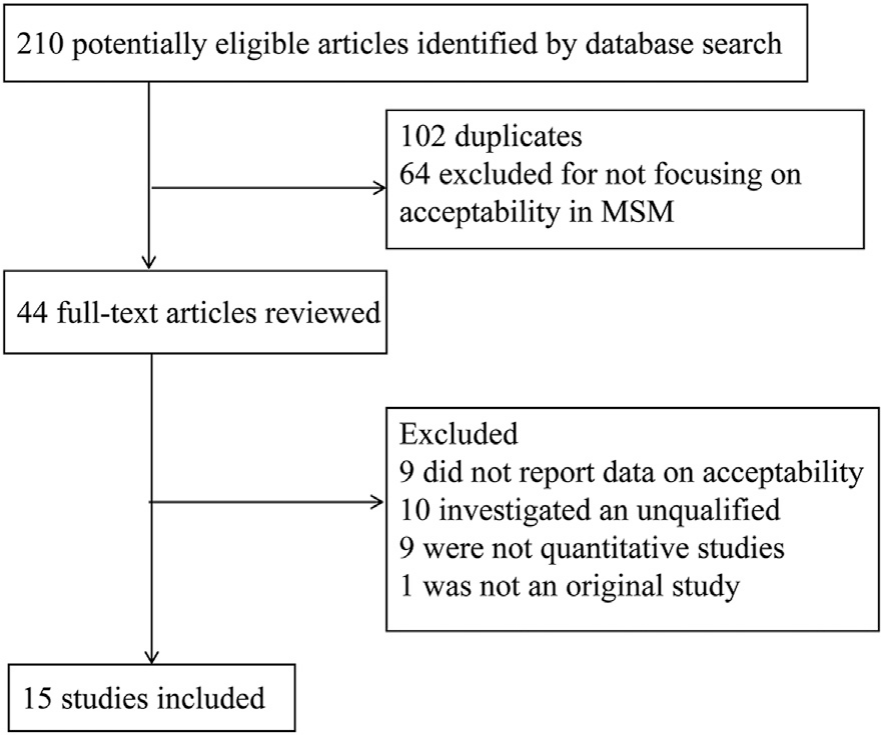
Flow diagram of the article selection. MSM, men who have sex with men.

Two investigators identified eligible articles independently. Disagreements between the investigators was resolved through discussion. The complete search process is shown in Figure 1.

### Outcome Definition

HPV vaccine acceptability among MSM, expressed through rates or scales, was the primary outcome. The factors associated with HPV vaccine acceptance were taken as the secondary outcome. The effect size in dichotomous outcomes was determined through summary OR with 95% CI values, and for continuous outcomes, SMD values.

### Data Extraction

We collected the following characteristics from each eligible study: Year of publication, author name, age range of sample, sample size, country, and rates or scales of acceptability. In studies with dichotomous outcomes, we extracted the number of people who accepted vaccination and did not accept vaccination in the two groups with or without factors associated with the acceptability. We extracted the mean, standard deviation (SD), and number of people who accepted and did not accept vaccination in the studies with continuous outcomes. The two investigators extracted data separately, and any dispute was resolved through discussion.

### Risk of Bias Assessment

To assess the quality of eligible studies, we used the quality evaluation tool for cross-sectional study recommended by the Agency for Healthcare Research and Quality (AHRQ), a checklist with 11 items. The 11th item of the scale was not applicable and therefore omitted. The remaining ten items are shown in Table 1.

**TABLE 1:** Quality assessment of included studies.

Studies	Item 1	Item 2	Item 3	Item 4	Item 5	Item 6	Item 7	Item 8	Item 9	Item 10	Bias
Simatherai et al. (2009)	Yes	Yes	Yes	Yes	No	No	No	No	No	Yes	High
Hernandez et al. (2010)	Yes	Yes	Yes	Yes	No	No	No	Yes	No	No	High
Reiter et al. (2010)	Yes	Yes	Yes	Yes	No	Yes	Yes	Yes	No	Yes	Moderate
Wheldon et al. (2011)	Yes	Yes	Yes	Yes	No	Yes	No	Yes	No	No	High
Rank et al. (2012)	Yes	Yes	Yes	Yes	No	Yes	No	Yes	No	Yes	Moderate
Sanchez et al. (2012)	Yes	Yes	Yes	Yes	No	No	No	Yes	No	No	High
Gutierrez et al. (2013)	Yes	Yes	Yes	Yes	No	No	No	Yes	No	No	High
Wang et al. (2013)	Yes	Yes	Yes	Yes	No	No	No	Yes	No	No	High
Zou et al. (2014)	Yes	Yes	Yes	Yes	No	No	No	Yes	No	No	High
Sadlier et al. (2016)	Yes	Yes	Yes	Yes	No	Yes	Unclear	Yes	No	No	High
Giuliani et al. (2016)	Yes	Yes	Yes	Yes	No	Yes	Yes	Yes	No	Yes	Low
Zou et al. (2016)	Yes	Yes	Yes	Yes	No	No	No	Yes	No	No	High
Nadarzynski et al. (2018)	Yes	Yes	Yes	Yes	No	Yes	Yes	Yes	No	No	Moderate
Li et al. (2019)	Yes	Yes	Yes	Yes	No	Yes	Yes	Yes	Yes	No	Low
Tian et al. (2019)	Yes	Yes	Yes	Yes	No	No	Unclear	Yes	Unclear	No	High

The 10 evaluation items are as follows.

1) Define the source of information (survey, record review).

2) List the inclusion and exclusion criteria for exposed and unexposed subjects (cases and controls) or refer to previous publications.

3) Indicate the time period used for identifying patients.

4) Indicate whether or not the subjects were consecutive, if not population-based.

5) Indicate if the evaluators of the subjective components of the study were masked to other aspects of the status of the participants.

6) Describe any assessments undertaken for quality assurance purposes, (e.g. test/retest of primary outcome measurements).

7) Explain the exclusion of any patient from the analysis.

8) Describe how confounding variables were assessed and/or controlled.

9) If applicable, explain how missing data were handled in the analysis.

10) Summarize the patient response rates and completeness of the data collection.

Some scholars do not recommend using the quality evaluation standard recommended by AHRQ in calculating the overall quality score because it may be misleading. We defined high-risk studies as those having two or more items that could increase the risk of bias among the 10 items. We defined low-risk studies as those having less than one item that could increase the risk of bias. A “YES” to the fifth item, which is contrary to others, can increase the risk(Yang et al., 2019).

### Statistical Analysis

Acceptability data were processed using the rescaling method and expressed in the hundred-mark system. We then performed a meta-analysis of the acceptability. We used Stata (v25) to process this part of the data.

We performed a meta-analysis of the studies that included factors associated with acceptability among MSM, using Review Manager (v5.3). The numbers of people who accepted and did not accept vaccination in the two groups with or without factors associated with the acceptability were used to calculate the summary OR and 95% CI. The mean, SD, and numbers of people who accepted and did not accept vaccination were used to calculate the SMDs.

### Assessment of Heterogeneity

Heterogeneity was assessed statistically using H index and I^2^ measurement. Multiple individual studies were considered to have heterogeneity when I^2^ > 50% or H > 1.5. We analyzed the source of heterogeneity by using sensitivity analysis, sub-group analysis, regression analysis. We chose the random effects model to aggregate SMDs and ORs on the condition of I^2^ > 50%. Otherwise, we used the fixed effects model.

### Sensitivity Analysis

We conducted sensitivity analysis to assess the robustness of the meta-analysis. We mainly compared the results, research quality, and experimental design of all included studies.

## Results

### Selection Process and Study Quality Evaluation

Our electronic database search yielded 210 articles. Finally, 15 articles with a total of 8,658 participants were included (Simatherai et al., 2009; Hernandez et al., 2010; Reiter et al., 2010; Wheldon et al., 2011; Rank et al., 2012; Sanchez et al., 2012; Gutierrez et al., 2013; Wang et al., 2013; Zou et al., 2014; Cummings et al., 2015; Giuliani et al., 2016; Sadlier et al., 2016; Zou et al., 2016; Nadarzynski et al., 2018; Li et al., 2019; Tian et al., 2019). The specific screening process is shown in Figure 1. After the review of titles and abstracts, we excluded 166 articles. According to a reading of the full text, we excluded nine articles because they did not report data on acceptability, ten articles because they investigated unqualified populations, nine articles because they were not quantitative studies, and one article because it was not an original study.

Of the included studies, five were from the United States, four from China, two from Australia, and one each from Canada, Iceland, Italy, and the United Kingdom. Most surveys were conducted in adult MSM, and four studies focused on young MSM. Eleven articles were published after 2011, when the US approved HPV vaccination for MSM. Table 1 lists the study characteristics and acceptability of HPV vaccines.

Regarding risk, 10 (62.5%), 4 (25%), and 2 (12.5%) studies were found to have high, medium, and low risk of bias. The assessment is listed in Table 1.

### Acceptability of HPV Vaccines Among MSM

The acceptability among the 15 studies was 50% (95% CI: 0.27–0.72). (Table 2). The I^2^ of the 15 studies was 99.9%, *p*＜0.001, which showed high heterogeneity. The sensitivity analysis showed that the result was stable. Then we respectively conducted subgroup analysis according to the region (*p* = 0.06), publication time (*p* = 0.53), and whether participants were informed of the vaccine price and efficacy (*p*＜0.05), which indicated that “informed of the vaccine price and efficacy” may be the source of heterogeneity. Regression analysis got the same result.

**TABLE 2:** Studies addressing human papillomavirus (HPV) vaccine acceptability, study characteristics, and risk of bias, ordered by publication year.

Author(s)	Year	Age (range, median, in years)	Sexual orientation/behaviour^b^ (%)	Sample size	Country	HPV vaccine acceptability
Simatherai et al. (2009)	2009	19–71, n/r	N/R	200	Australia	47%
Hernandez et al. (2010)	2010	N/R, n/r	N/R	88	United States	75%
Reiter et al. (2010)	2010	18–59, n/r	Gay (77.1%), bisexual (22.9%)	306	United States	74%
Wheldon et al. (2011)	2011	18–29, n/r	Gay (83.3%), bisexual (16.2%)	179	United States	36%
			Unclear (0.5%)			
Rank et al. (2012)	2012	19–83, M = 33	Gay (80.4%)	1,169	Canada	67%
			Bisexual (11.0%), other (8.6%)			
Sanchez et al. (2012)	2012	17–62, M = 25	N/R	116	United States	86.2%
Gutierrez et al. (2013)	2013	13–21, M = 18	N/R	41	United States	2.6^b^
Wang et al. (2013)	2013	18–60, n/r	Homosexual (86.7%)	542	Hong Kong, China	29.2%
			Bisexual/heterosexual/uncertain (13.3%)			
Zou et al. (2014)	2014	16–20, M = 19	N/R	200	Australia	30%
Sadlier et al. (2016)	2016	18–60, n/r	N/R	302	Ireland	31%^c^
Giuliani et al. (2016)	2016	29–46, M = 36	N/R	296	Italy	31.4%^c^
Zou et al. (2016)	2016	N/R, n/r	N/R	196	China	18.8%
Nadarzynski et al. (2018)	2018	14–63, M = 22	Gay (93%), bisexual (5%)	1,508	United Kingdom	83%
			Homosexual (74.5%)			
Li et al. (2019)	2019	N/R, 31^a^	Heterosexual (1.1%)	3,057	China	2.5%
			Bisexual (20.1%)			
			Not sure (4.3%)			
			Gay (95.0%)			
Tian et al. (2019)	2019	N/R, n/r	Bisexual/heterosexual (5.0%)	458	China	71.8%

^a^ average age.

^b^ Measured on a five-point scale, where 1 = weak, 3 = neutral, 5 = strong.

^c^ Acceptability regardless of vaccine price.

N/R = not reported.

### Factors Associated With Acceptability of HPV Vaccines Among MSM

The meta-analysis included seven articles (*n* = 4,200). The data we collected supported the meta-analysis of four aspects: socio-demographic characteristics, behavioral indicators, HPV education, and HPV risk perception. Table 3 gives the effect size, H value, and I^2^ value of the influence factors under these four themes on acceptability (Supplementary Material).

**TABLE 3:** Meta-analysis of correlates of human papillomavirus (HPV) vaccine acceptability among MSM.

Theme(s)	Factor(s)	Number of studies	Effect size (95% CI)	H	Between-study variability, I^2^
Socio-demographic characteristics	Educational level (college or higher degree)	5	1.62 [1.35, 1.95], *p* < 0.001	1.2 [1.0.1.9]	26% [0,71]
	In a relationship or married	5	1.08 [0.87, 1.35], *p* = 0.483	1.0 [1.0.2.2]	0% [0,79]
	HIV infection	3	1.10 [0.83, 1.47], *p* = 0.49	1.0 [1.0.3.1]	0% [0,90]
	Sex identity (gay vs. bisexual)	5	1.17 [0.79, 1.75], *p* = 0.041	1.6 [1.0.2.6]	60% [0,85]
Behavioral indicators	Disclosure of sexual orientation to HCPs	2	2.38 [1.47, 3.86], *p* < 0.001	–	80%
	Vaccination for hepatitis A or B at least one dose	3	2.10 [1.42, 3.10], *p* < 0.001	1.8 [1.0.3.3]	68% [0,91]
	Ever diagnosed with an STD	4	1.91 [0.94, 3.86], *p* = 0.072	2.6 [1.6.4.0]	85% [62,94]
HPV education	Heard of HPV	4	1.85 [1.21, 2.83], *p* = 0.005	2.0 [1.2.3.3]	74% [28,91]
	HPV knowledge	3	0.28 [0.16, 0.39], *p* < 0.001	1.3 [1.0.2.3]	38% [0,81]
	Heard of HPV vaccine	2	1.23 [0.76, 1.99], *p* = 0.41	–	32%
HPV risk perception	Perceived susceptibility to HPV infection	2	0.31 [0.11, 0.50], *p* = 0.002	–	22%
	Perceived severity of HPV-related disease	3	0.40 [0.28, 0.51], *p* < 0.001	1.0 [1.0.3.1]	0% [0,90]

#### Socio-Demographic Characteristics

Having a college degree or above could promote MSM to accept HPV vaccine. We did not find a statistically significant relation between acceptability and HIV status, sex identity or relationship status.

#### Behavioral Indicators

Disclosure of sexual orientation and vaccination for hepatitis A or B at least one dose could both promote acceptance of HPV vaccine among MSM. We did not find a statistically significant relation between having a previous diagnosis of an STD and acceptability.

#### HPV Education

People who know more HPV knowledge or heard of HPV were more likely to vaccinate HPV vaccine. We did not find a statistically significant relation between HPV vaccine awareness and acceptability.

#### HPV Risk Perception

Perceived susceptibility to HPV infection and perceived severity of HPV-related disease could both promote the acceptance of HPV vaccines among MSM.

#### Heterogeneity Testing and Subgroup Analyses

The meta-analysis showed high heterogeneity among the studies in terms of gender identity. After sensitivity analysis, we eliminated the study by Wang et al. and the summary estimates changed (OR = 1.47, 95% CI: 1.12–1.94). Wang et al. recruited participants through saunas and bars, which may have led to higher consistency. We performed a subgroup analysis based on whether the articles’ publication time was before 2015 and found that this can explain the source of heterogeneity of the two associated factors (previous diagnosis of an STD and HPV vaccine awareness).

Besides, disclosure of sexual orientation to HCPs and vaccination for hepatitis A or B at least one dose all had high heterogeneity. Since the studies in these two aspects only included no more than three studies, subgroup analysis cannot be performed. Based on this, we chose the random-effects model for meta-analysis.

## Discussion

This meta-analysis showed that the MSM population has a moderate acceptance of HPV vaccine, which showed a wide range of acceptability. Although the acceptance in our results was lower than that of the men (50.4, SD: 21.5) in Newman et al. (Newman et al., 2013), the results of acceptability in the two studies are similar and both indicate moderate acceptance. Some investigations did not exclude people who have been vaccinated, which is different from the inclusion and exclusion criteria of this study. As such, the differences in outcomes may be attributed to the differences in inclusion criteria. Paul et al. found that 8% of participants who have not been vaccinated are still willing to pay $400 for HPV vaccination among women who have sex with women (Reiter et al., 2020), which means there is still a big gap between vaccine acceptance and target coverage. As mentioned above, the vaccination rate should be above 70% to achieve the protective effect. The current population has only moderate acceptance, which is far from our goal. At the same time, we should find ways to eliminate the barriers to vaccination. This study focuses on the associated factors affecting acceptance, and how to improve acceptance will be discussed further below.

The meta-analysis indicated the influence of education level, behavior indicators, HPV-related knowledge, and risk perception on HPV vaccine acceptability for MSM. Acceptability is associated with educational level (having a college or higher degree). People with higher education level have a stronger sense of self-management and are more receptive to new things (Wang et al., 2013). Meanwhile, it is more difficult for low-education people to accept the HPV vaccine, which means special attention should be paid to this group. In China’s rural or mountainous areas, education, medical care, and material conditions are often limited, which leads to lower acceptance of HPV vaccines than urban residents (Tian et al., 2019). Therefore, staff should go to remote areas to conduct popular science and health education. Besides, when educating them, vaccination campaigners should ensure the easy comprehensibility of teaching materials. Besides, hospitals should work with health authorities to strive for more preferential policies for residents in remote areas to conduct HPV testing. At the same time, we should strengthen general practitioners’ training in remote areas to ensure that they can do an excellent job in educating patients about HPV.

People who disclosed their sexual orientation to HCPs were more willing to avail of the HPV vaccine. HCP recommendation can promote the acceptability of HPV vaccine among MSM, as confirmed in male and female populations (Brewer and Fazekas, 2007; Newman et al., 2013). The acceptability of the HPV vaccine reaches up to 89% among MSM, if offered by HCPs (Nadarzynski et al., 2018). However, attention should be given to the fact that MSM are stigmatized when seeking treatment for HPV and anal cancer. Given the stress of being in the minority, they cannot easily disclose their sexual orientation to the doctor and seek help. Health professionals should devote attention to reducing MSM stigma, which is a challenging problem (Sauvageau and Dufour-Turbis, 2016). STDs department can cooperate with community-based organizations (CBO), and CBO staff can help MSM eliminate the shame of sexual minorities through peer education, which can increase MSM’s trust in doctors at the same time.

Meanwhile, people without self-protection behavior have difficulty accepting HPV vaccines. Rank et al.(Rank et al., 2012) and Wang et al. (Wang et al., 2013). indicated that engaging in unprotected anal sex can reduce the acceptability of the HPV vaccine. Studies have also confirmed that people with more sex partners are less likely accept the HPV vaccine (Reiter et al., 2010; Rank et al., 2012; Zou et al., 2014; Nadarzynski et al., 2018). In addition, studies have found that HPV combined with *Chlamydia* or *Mycoplasma* infection can increase the risk of anal intraepithelial neoplasia. These findings point to a good method for strengthening vaccine acceptability: HPV vaccine education may be combined with STD education to educate the target population effectively. Doctors should conduct regular HPV education for STD patients. STDs departments can make their HPV brochures for patients who come for diagnosis and treatment of STDs.

Understanding HPV and HPV-related risks can increase acceptance. However, people generally lack knowledge on HPV and HPV vaccines. Wang et al.(Wang et al., 2013). found that people who perceive the harms of HPV-related disease among MSM are more willing to accept HPV vaccines. Thus, When carrying out HPV education, emphasis should be placed on the high incidence of HPV-related disease, and the harm of HPV-related disease to the body should be explained in detail (Newman et al., 2013).

The level of the knowledge also varies among different populations. The knowledge range tends to be lower in men (31%) than in women (31–65%), whereas that among adolescents is at 20.0%, which needs to be improved urgently (Gamaoun, 2018). A study in Urumqi, China (Tian et al., 2019), showed that local residents have a better understanding of HPV knowledge compared with non-local residents, most of whom came from rural or county towns around Urumqi. This may be due to the fact that Urumqi residents have more information sources than those who come to Urumqi to work. In addition, when promoting HPV knowledge, campaigns often start with local residents. Thus, there is a strong imbalance in the knowledge on HPV and HPV risk between urban and non-urban residents, and between men and women. This is undoubtedly an obstacle to the acceptance of HPV vaccines.

The meta-analysis also revealed that there is no statistically significant relation between HIV status and acceptability, which was beyond our expectation. We formed two hypotheses regarding this matter. First, HIV infection will increase people’s self-care awareness and prompt them to seek STD knowledge, which will increase their acceptance of HPV vaccines. Second, considering that AIDS cannot be cured currently, people may be more unscrupulous in their sexual behavior. In other words, the acceptance of the HPV vaccine among HIV- positive MSM depends on their attitude after they are infected with HIV. If HIV infection makes MSM pay more attention to their health, they will be more willing to accept the HPV vaccine. Otherwise, they will resist the HPV vaccine. The result once again suggests the importance of peer education and doctor education for MSM. Doctors and CBO staffs need to guide and encourage MSM especially after they have just been infected with HIV. HIV- positive MSM comprise a high-risk group for HPV-related disease. Helping them get an active attitude toward the HPV vaccine is of great significance for improving vaccines’ coverage and protective effects.

In this meta-analysis, there were unavoidable limitations in several aspects that should be further considered. Compared with HPV vaccine knowledge, the structural barriers (such as the cost of the vaccine) can influence acceptability to a greater degree, as identified among men and girls by Newman et al. (Newman et al., 2013). Besides, in some of the results with high heterogeneity, we have not performed subgroup analysis because of limited data. Some of our data is highly heterogeneous, which shows that more research is needed to verify the conclusion.

Another limitation is that the included studies were all cross-sectional in design. In future, more rigorously designed cohort studies are needed to confirm our conclusions. Although we included research from various countries, the data of developing countries were only from China. Besides, we potentially excluded papers written in languages apart from English and documents outside these three databases or published in the gray literature. Also, the study doesn’t include investigations that focus on the acceptability of the HPV across populations. Therefore, the conclusions of this article should be considered with caution.

## Conclusion

The acceptability of the HPV vaccine among MSM is at a moderate level. It is necessary to strengthen HPV vaccine education in the MSM population. Attention should be paid to people with low education levels, non-urban residents, and those with risky behavior factors. Education should emphasize the high incidence of HPV-related disease and their harm to health.

## Data Availability

Publicly available datasets were analyzed in this study. This data can be found here: Cuie Liu, liu2020@ccmu.edu.cn; Xiaojie Huang, huangxiaojie78@126.com.
