# Supplementary figures and images for: Improving the Acceptability of Human Papillomavirus Vaccines Among Men Who Have Sex With Men According to the Associated Factors: A Systematic Review and Meta-analysis
Source: Front Pharmacol. 2021 Mar 24;12:600273. doi: 10.3389/fphar.2021.600273 (PMC8044753; doi:10.3389/fphar.2021.600273)

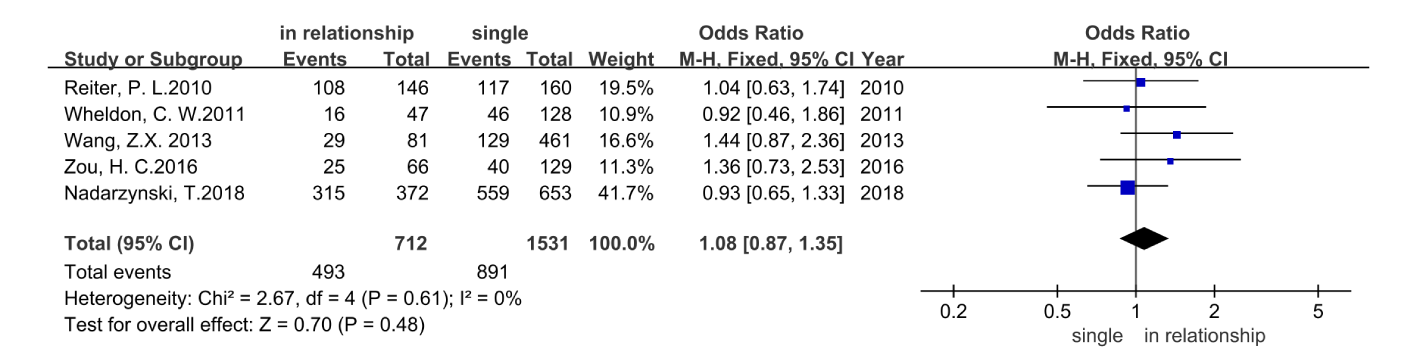

Supplement: Supplementary file 1 [file datasheet1.zip › Supplementary materials/Image 1.TIFF]

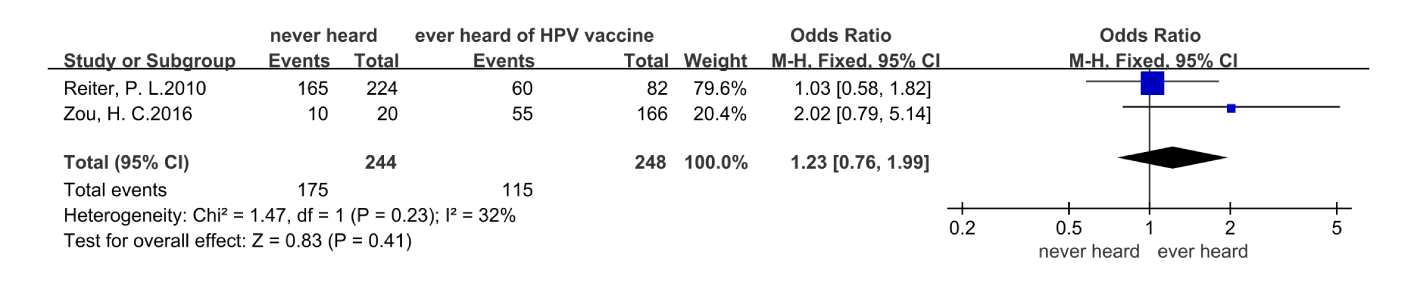

Supplement: Supplementary file 1 [file datasheet1.zip › Supplementary materials/Image 10.TIFF]

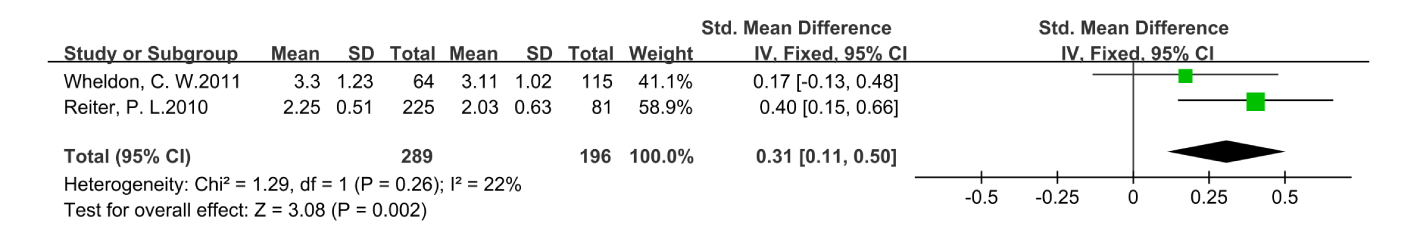

Supplement: Supplementary file 1 [file datasheet1.zip › Supplementary materials/Image 11.TIFF]

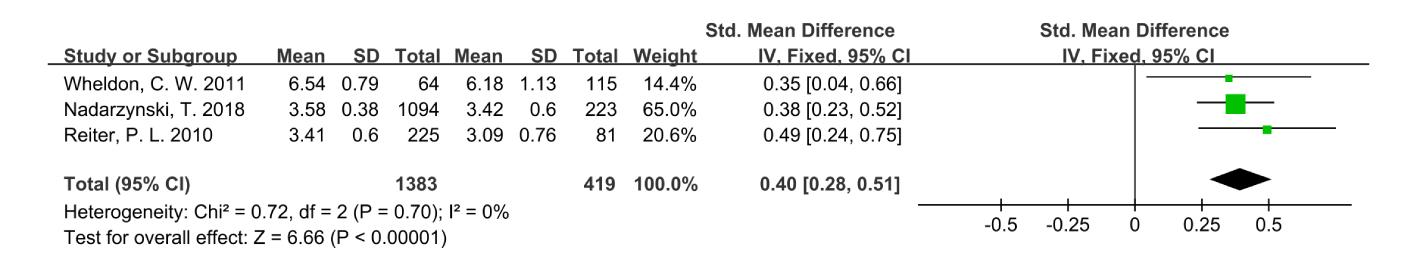

Supplement: Supplementary file 1 [file datasheet1.zip › Supplementary materials/Image 12.TIFF]

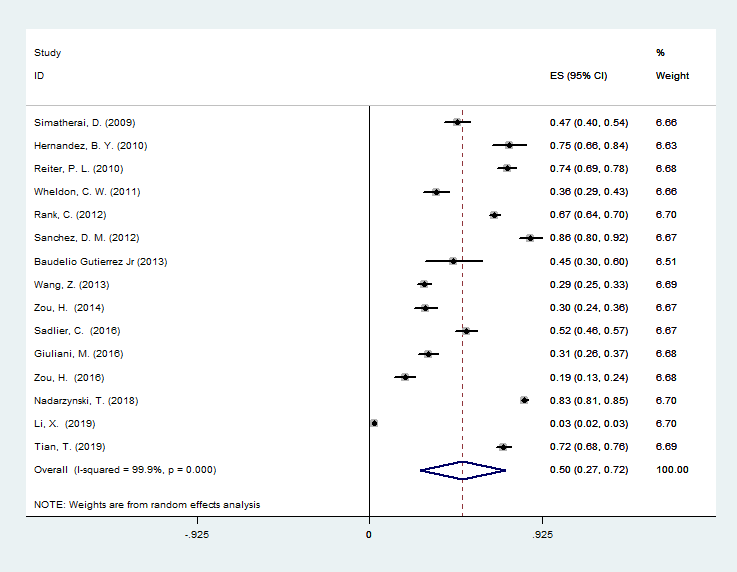

Supplement: Supplementary file 1 [file datasheet1.zip › Supplementary materials/Image 13.tif]

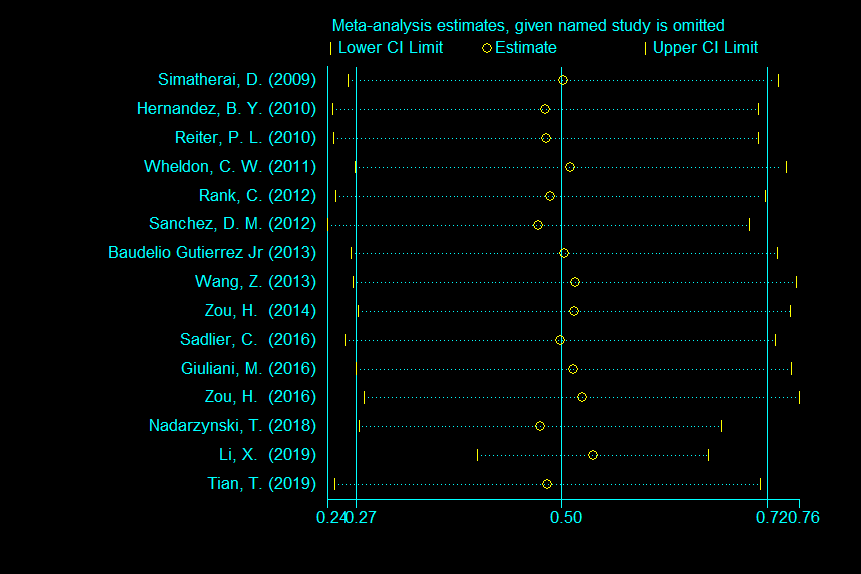

Supplement: Supplementary file 1 [file datasheet1.zip › Supplementary materials/Image 14.tif]

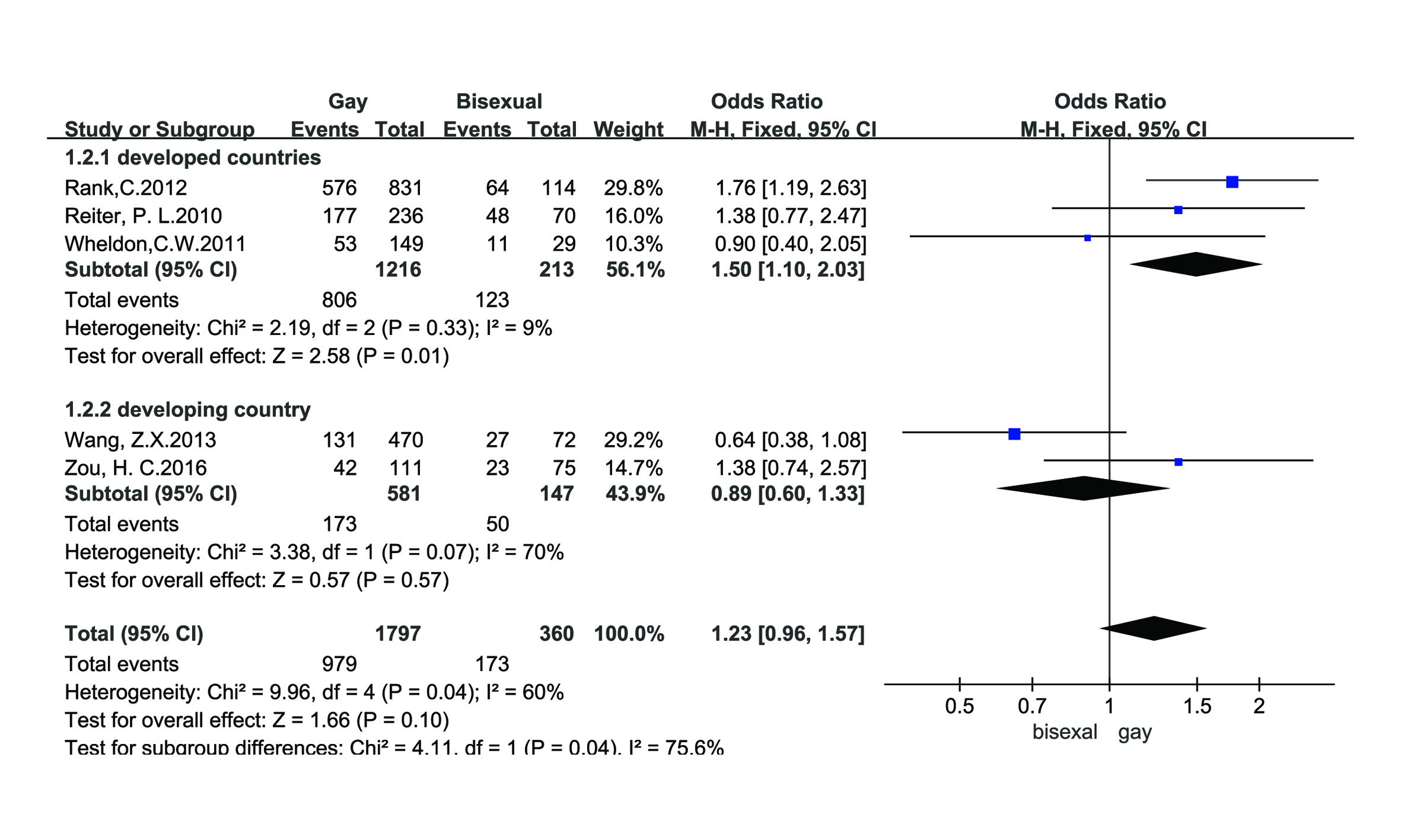

Supplement: Supplementary file 1 [file datasheet1.zip › Supplementary materials/Image 15.jpg]

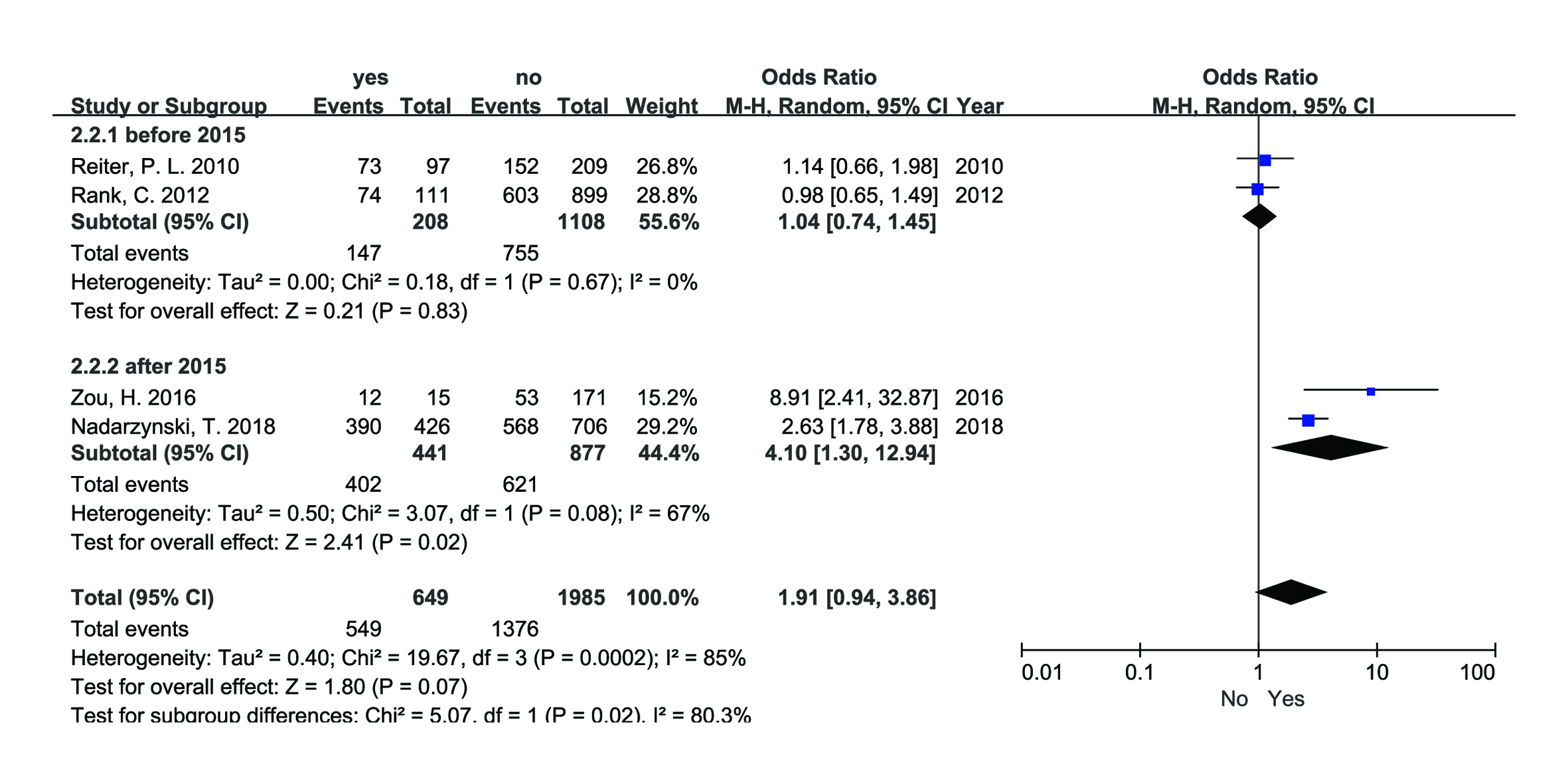

Supplement: Supplementary file 1 [file datasheet1.zip › Supplementary materials/Image 16.jpg]

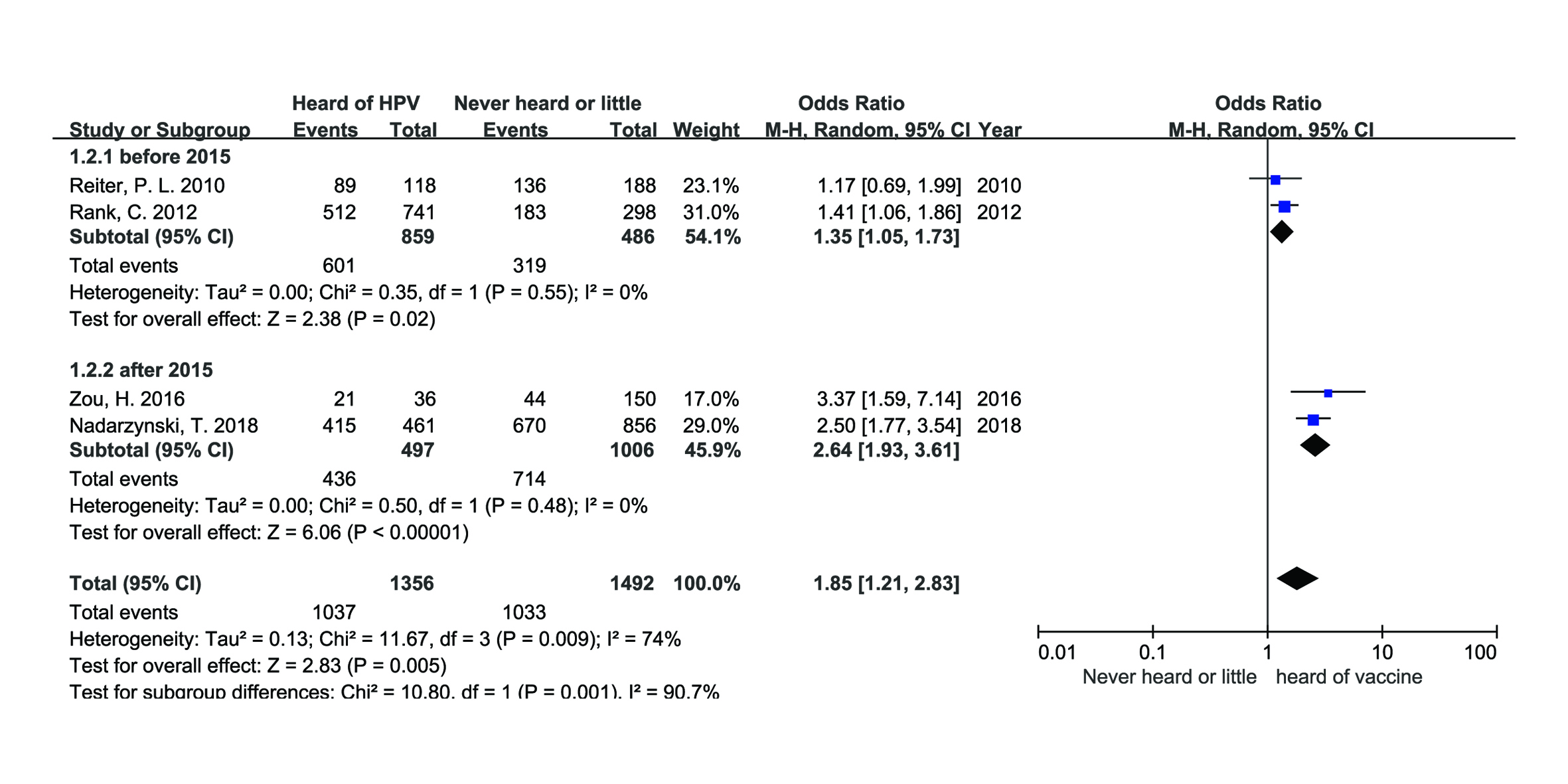

Supplement: Supplementary file 1 [file datasheet1.zip › Supplementary materials/Image 17.jpg]

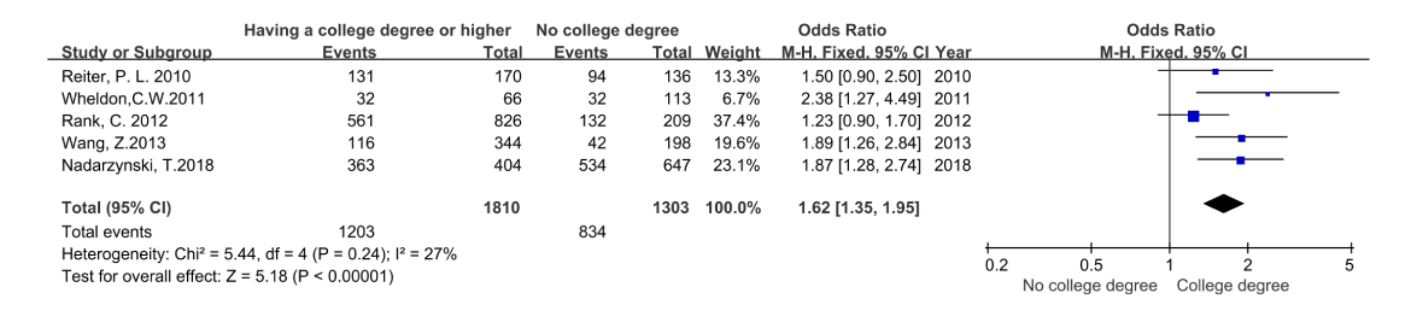

Supplement: Supplementary file 1 [file datasheet1.zip › Supplementary materials/Image 2.TIFF]

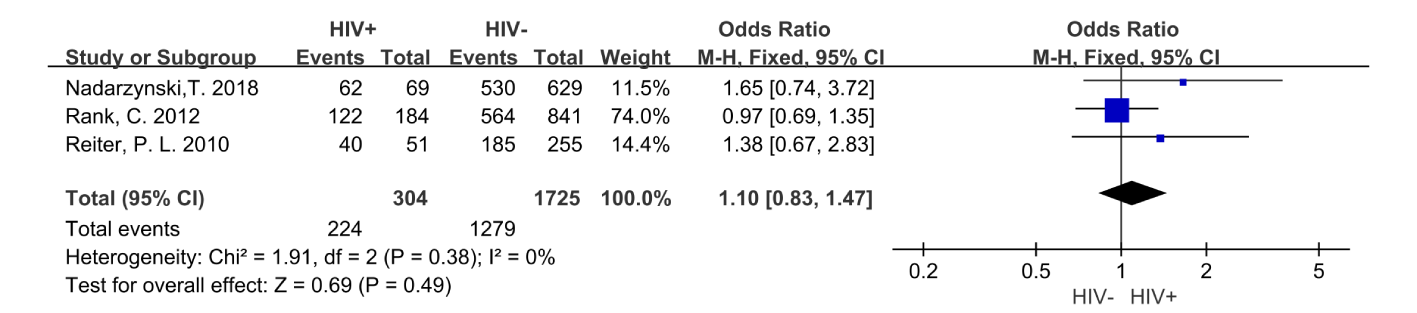

Supplement: Supplementary file 1 [file datasheet1.zip › Supplementary materials/Image 3.TIFF]

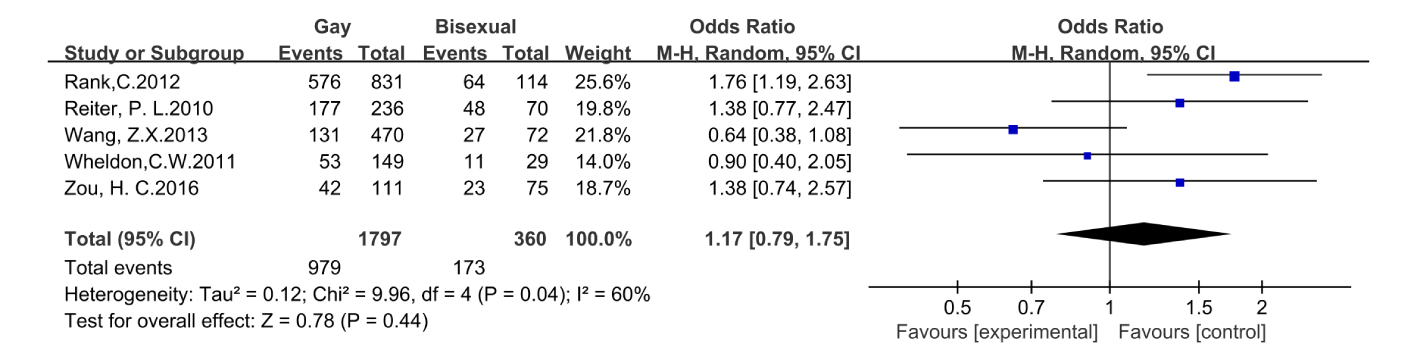

Supplement: Supplementary file 1 [file datasheet1.zip › Supplementary materials/Image 4.TIFF]

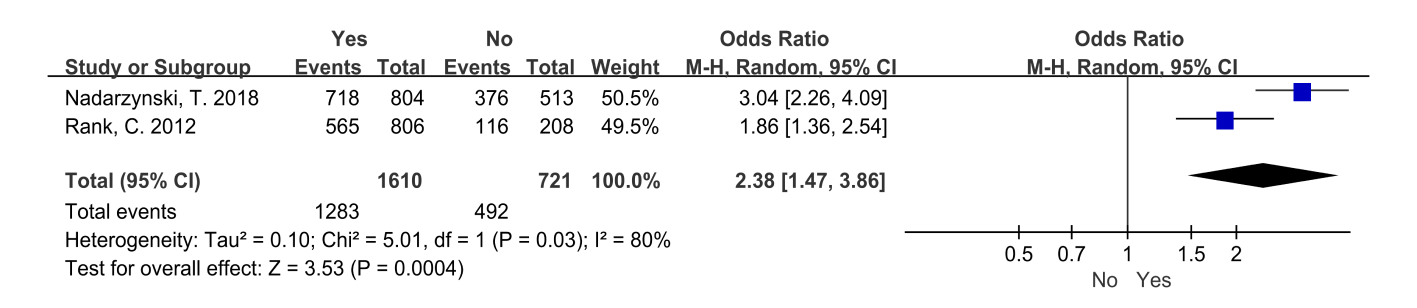

Supplement: Supplementary file 1 [file datasheet1.zip › Supplementary materials/Image 5.TIFF]

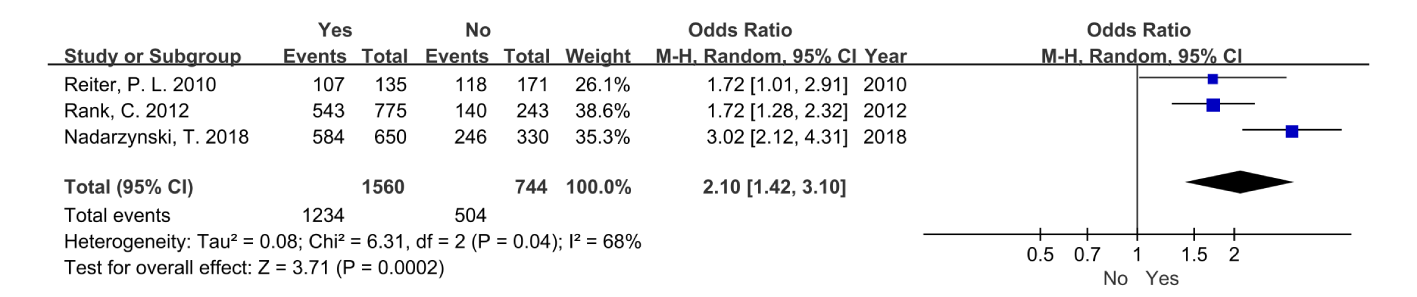

Supplement: Supplementary file 1 [file datasheet1.zip › Supplementary materials/Image 6.TIFF]

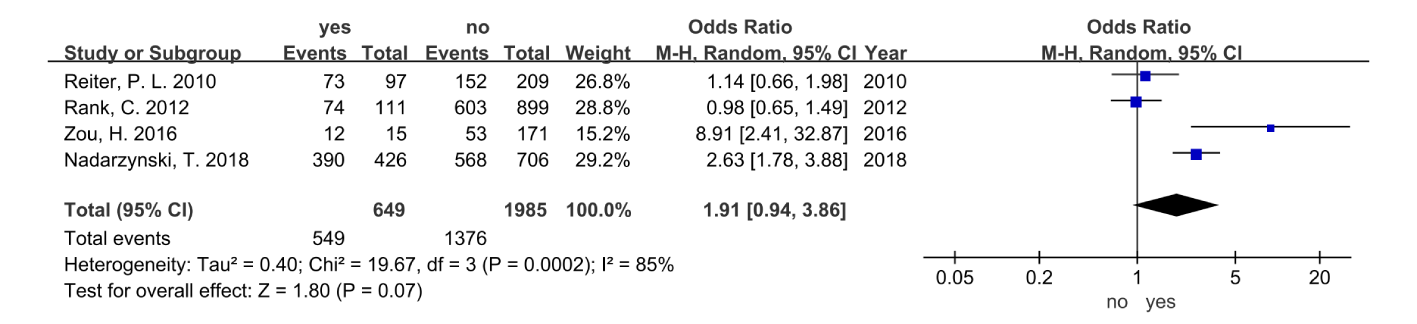

Supplement: Supplementary file 1 [file datasheet1.zip › Supplementary materials/Image 7.TIFF]

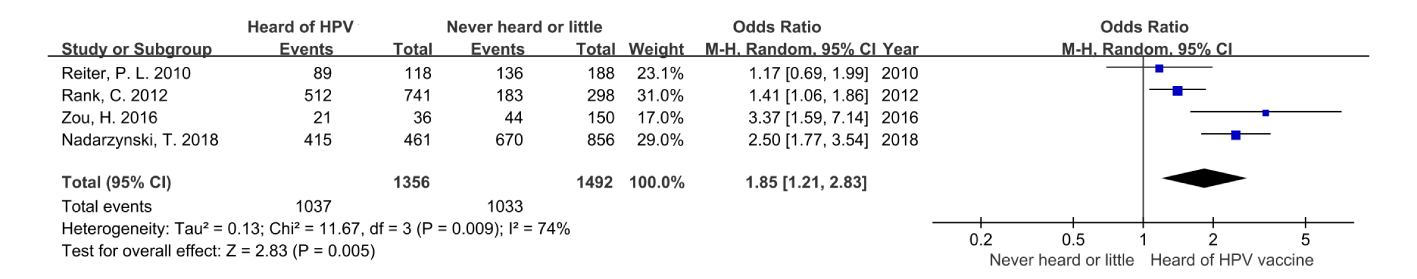

Supplement: Supplementary file 1 [file datasheet1.zip › Supplementary materials/Image 8.tif]

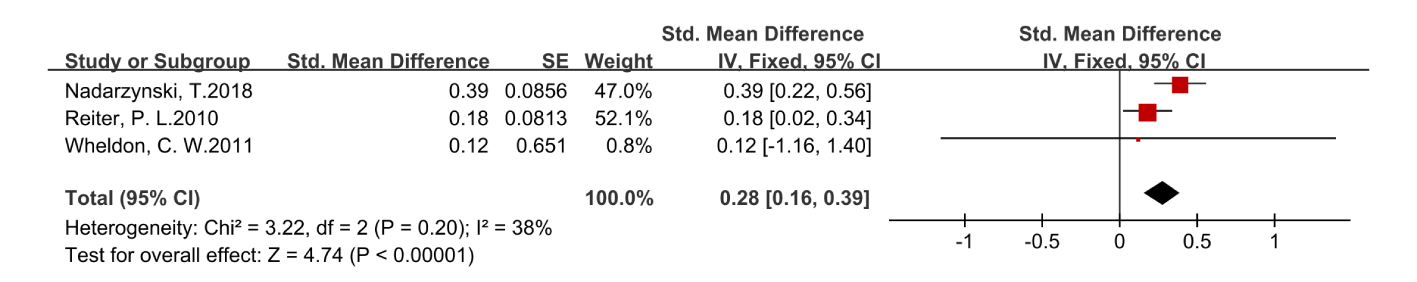

Supplement: Supplementary file 1 [file datasheet1.zip › Supplementary materials/Image 9.TIFF]
